# “King of the Riverside”, a multi-proxy approach offers a new perspective on mosasaurs before their extinction

**DOI:** 10.1186/s40850-025-00246-y

**Published:** 2025-12-12

**Authors:** Melanie A. D. During, Nathan E. Van Vranken, Clint A. Boyd, Per E. Ahlberg, Suzan J. A. Warmerdam-Verdegaal, Jeroen H. J. L. Van der Lubbe

**Affiliations:** 1 Evolutionary Biology Centre, Department of Organismal Biology, Uppsala University, Uppsala, Sweden; 2 Department of Earth Sciences, Faculty of Science, Geochemistry & Geology Cluster, Vrije Universiteit Amsterdam, Amsterdam, Netherlands; 3 Biological Environmental Technology, Eastern West Virginia Community and Technical College, Moorefield, WV USA; 4 Fossil Resource Protection Program, North Dakota Geological Survey, Bismarck, ND USA

**Keywords:** Mosasaurs, Western interior seaway, Isotope analysis

## Abstract

The discovery of a mosasaurine tooth (NDGS 12217) in the Hell Creek Formation, Morton County, North Dakota, adds to the growing evidence that mosasaurs, traditionally considered marine reptiles, could inhabit freshwater environments. This tooth was found in a context devoid of other marine taxa, suggesting the individual may have ventured into freshwater environments. The morphology of the tooth supports a conservative referral to the tribe Prognathodontini, and stable isotope analyses on carbonate and phosphate fractions of tooth enamel, along with ^87^Sr/^86^Sr ratios, indicate a freshwater origin. These findings challenge the notion of mosasaurs as exclusively marine predators, highlighting their potential ecological flexibility during the Late Maastrichtian. This study employs a multi-proxy approach to compare the tooth’s isotopic signatures with other taxa from the same and nearby formations (including teeth of other mosasaurines), providing insights into the environmental transitions of the Western Interior Seaway (WIS) towards the end of the Cretaceous. This comprehensive approach aims to provide deeper insights into the behavior, ecology, and potential migratory patterns of mosasaurs beyond their known marine habitats, enhancing our understanding of their adaptation to rapidly changing environments during the Late Cretaceous.

**Supplementary Information:**

The online version contains supplementary material available at 10.1186/s40850-025-00246-y.

## Introduction

Mosasaurs were apex marine predators that diversified during the Late Cretaceous, dominating the seas and occupying a variety of marine niches [1]. Within Mosasauridae, three subfamilies are recognized: Mosasaurinae, Plioplatecarpinae, and Tylosaurinae, each characterized by distinct morphological adaptations that allowed them to exploit different ecological opportunities [1, 2]. Though mainly associated with shallow seas, mosasaur remains in estuarine and freshwater environments challenge their traditional strictly marine classification [3–5].

**Fig. 1:**
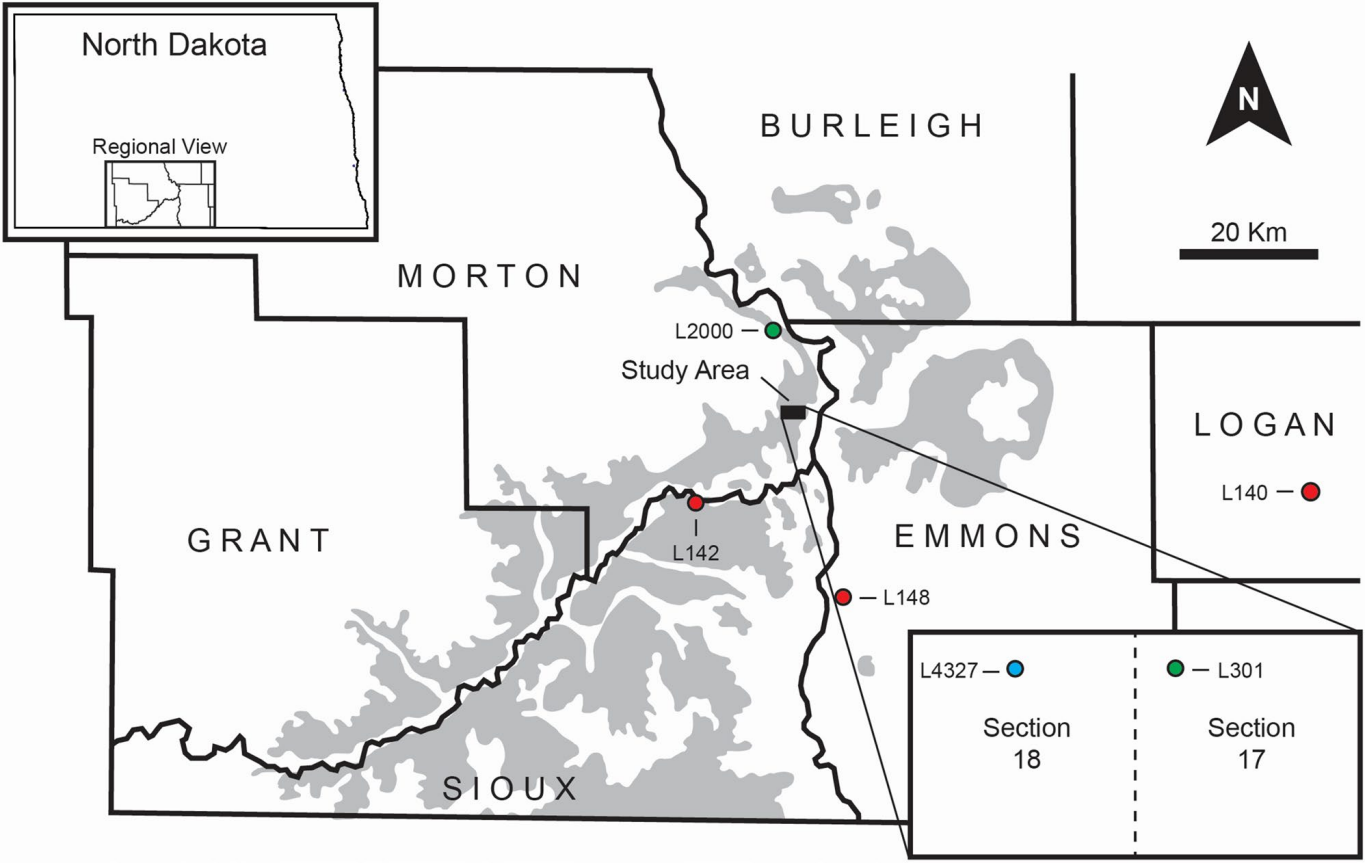
Study locations in south-central North Dakota, with the rectangle marking the study area and an inset showing site NDGS L4327 (blue) relative to NDGS L301 (green) [5]. Fox Hills Formation localities in this study are marked red (L140, L142, and L148) and Breien Member localities (L2000) green. Hell Creek Formation exposures are shaded

In 2022, a mosasaurine tooth crown (NDGS 12217) was discovered in a multi-taxon vertebrate fossil locality in the Hell Creek Formation, Morton County, North Dakota (Figure 1). This site is notable for its lack of marine taxa, with fossils dominated by terrestrial and freshwater species. The discovery of NDGS 12217 raises questions about mosasaurs potentially inhabiting freshwater environments seasonally or as part of a broader ecological range. Previous mosasaurine material from this region has been found in stratigraphically lower, brackish deposits [5], but NDGS 12217 was recovered from a carbonaceous mudstone that is stratigraphically higher, suggesting it represents a different ecological setting.

**Fig. 2:**
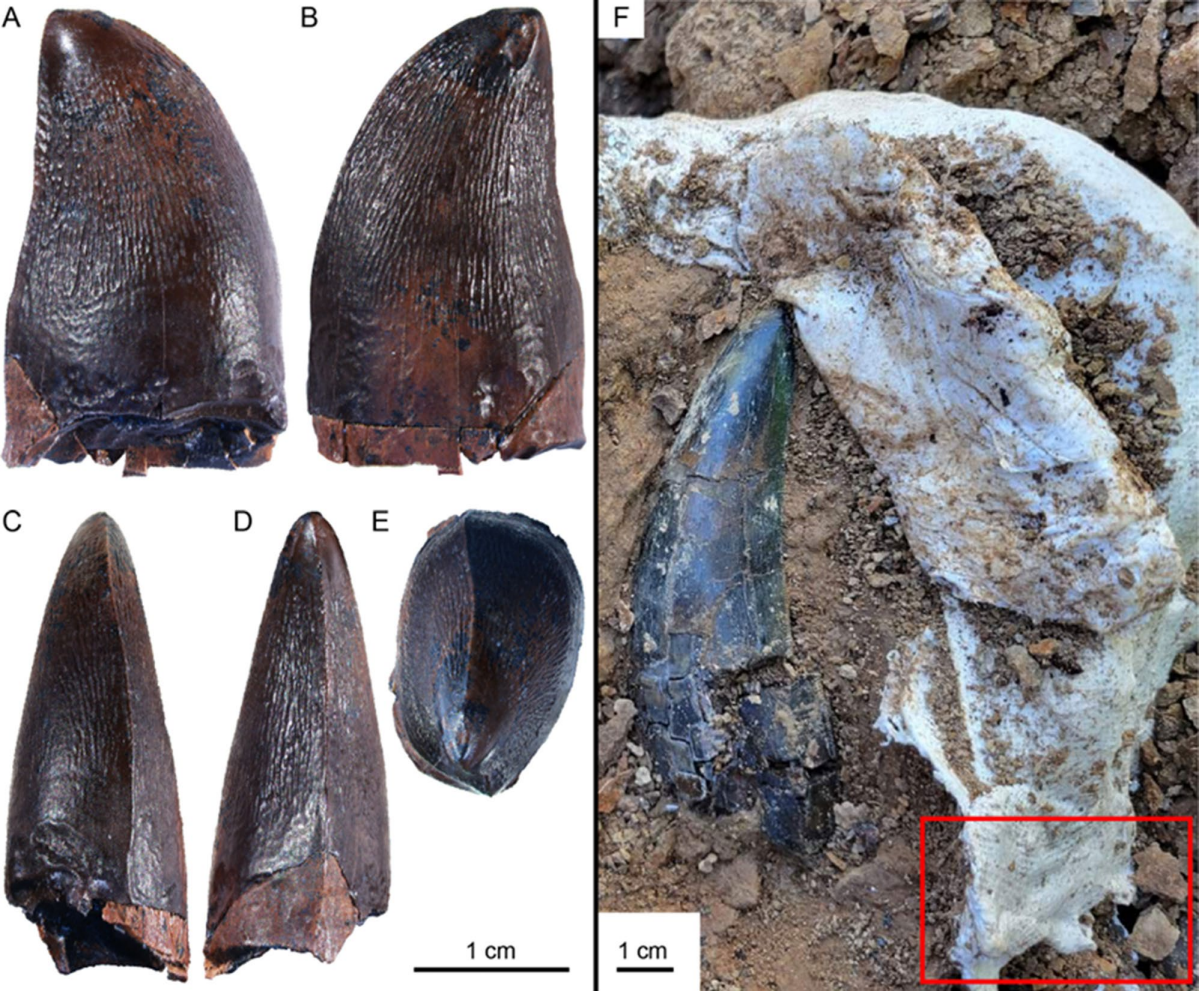
Tooth NDGS 12217 (Prognathodontini indet.) from the Hell Creek Formation, shown in lingual (**A**), labial (**B**), anterior (**C**), posterior (**D**), and occlusal (**E**) views. Image (**F**) shows the recovery location (red box) relative to a *Tyrannosaurus rex* tooth (NDGS 15125) at NDGS L4327

The tooth shows no signs of transport or reworking, indicating the individual may have lived in the Hell Creek freshwater paleoenvironment. (Figure 2). The tooth’s morphology, particularly its fine veined texture and lack of serrations, suggests it belongs to the tribe Prognathodontini, known for its robust dentition and broad distribution [1]. The mosasaur material previously reported from a nearby locality within the Breien Member may represent the same taxon which may indicate an estimated maximum length of approximately 11m [5].

The mosasaurine tooth (NDGS 12217) was found in a brown carbonaceous mudstone bed above a fine-grained sandstone in the Hell Creek Formation at locality NDGS L4327, Morton County, North Dakota, 1,090 kilometres west of the site where the ‘Breien Mosasaur’ was discovered [5] (Figure 1). The mudstone contains a palaeosol at the upper contact, rich in sesquioxide nodules, indicating a period of non-deposition before being overlain by sandstone [6]. Vertebrate fossils are concentrated in the lower metre of the carbonaceous mudstone bed that varies from ~ 1,5 to 3 m thickness at NDGS L4327.

The site is interpreted as a floodplain near a stream where terrestrial carcasses accumulated and were then scavenged, disarticulated, and damaged. Most vertebrate fossils in the mudstone are disarticulated and randomly distributed, showing minimal signs of subaqueous transport. No taphonomic evidence has been found for long-distance transport. Many fossil bones, however, do show pre-depositional breakage and feeding traces (e.g., scores and pits [6–8]), though trace makers are often hard to identify. Shed teeth from crocodylians (e.g., *Borealosuchus*, *Brachychampsa*) and theropods (e.g., *Archeroraptor*, *Tyrannosaurus*) are mixed with bones, including NDGS 12217 found near a *Tyrannosaurus* tooth (NDGS 15125), a *Brachychampsa* maxilla (NDGS 18199), and *Edmontosaurus* remains. The locality (NDGS L4327), in a displaced slump block above the Breien Member, suggests post-transgression deposition near the eastern edge of the Hell Creek Formation, close to the WIS shoreline. The absence of the Cantapeta Tongue [9] supports this distinct stratigraphic position.

Previous studies suggest some mosasaurids found on differing nodes of divergence among their family tree may have inhabited freshwater environments. For instance, a Canadian *Plioplatecarpus* sp. [estimated length 5m] has limb morphology potentially suited for shallow, constrained habitats [3]. Likewise, the Hungarian *Pannoniasaurus inexpectatus* [estimated length 6m] shows pelvic anatomy and limb features suggesting amphibious lifestyles [4]. *Goronyosaurus nigeriensis* [estimated length, 5m] developed a crocodile-like skull to navigate the coastal and estuarine environments of the Dukamaje Basin [10]. These mosasaur examples are thus notably small when compared to both the Breien mosasaur [5] as well as what can be expected for NDGS 12217. Geochemical evidence supports freshwater habitats for these smaller mosasaurids, with strontium isotopes indicating mosasaurs lived in freshwater environments in Hungary and oxygen isotopes suggesting freshwater excursions in the northern WIS [11, 12]. However, these methods individually do not preclude potential post-mortem displacement or diagenetic alteration affecting the results. To overcome these uncertainties we measured both isotopic compositions, and added stable carbon isotopic analyses, which combined, likely exclude any single diagenetic alternation pathway, but would reveal the in-vivo composition of the organism.

This study employs a multi-proxy approach, combining stable carbon and oxygen isotope analyses on the structural carbonate (δ^13^C and δ^18^O_Sc_), stable oxygen isotope analysis on the phosphate (δ^18^O_P_) and strontium isotope ratios (^87^Sr/^86^Sr) to investigate the habitat preference of the mosasaur represented by NDGS 12217. By comparing these results with those from presumed marine, brackish, and freshwater taxa from geographically and temporally equivalent deposits, we aim to shed light on the environmental context of this specimen and its implications for understanding mosasaur ecology during the Late Cretaceous. Our analyses, furthermore, allow for comparison to temperature and salinity records of the WIS [13, 14] and enable us to assess the salinity of WIS towards the end of the Cretaceous.

## Materials and methods

### Institutional abbreviations

The mosasaurine tooth (NDGS 12217), along with a *T. rex* tooth (NDGS 15125), a crocodylian maxilla with teeth (NDGS 18199), and a hadrosaurine tooth (NDGS 18198), were recovered from a carbonaceous mudstone bed in the Hell Creek Formation at locality NDGS L4327 in Morton County, North Dakota in 2022.

To compare with other time-equivalent WIS environments, additional analyses were conducted on a diverse selection of available specimens from the marine Fox Hills Formation (Figure 1): an ammonite fragment (NDGS 18190) and a shark tooth (NDGS 18189) from the Timber Lake Member in Logan County (NDGS L140); a crocodilian tooth (NDGS 20301) and a mosasaur tooth (*Mosasaurus dekayi* NDGS 20300) from the Timber Lake Member in Emmons County (NDGS L148); and a mosasaur tooth (*?Plioplatecarpus* NDGS 20303) from the Colgate Member in Sioux County (NDGS L142). Three specimens from the marine-to-brackish Breien Member (Figure 1) were also analyzed: a shark tooth (NDGS 5612) from the “Breien Mosasaur” locality (L301); a crocodylian tooth (NDGS 20304) and a *T. rex* tooth (NDGS 20305) from Morton County (NDGS L2000).

### Measurements

Measurements were taken with a Mitutoyo CD-6 micrometer and rounded to the nearest 0.1 mm. The mosasaur tooth (NDGS 12217) was imaged using an Olympus SZX16 microscope and Olympus Stream Essentials software. Multiple images of the tooth crown were captured and stitched together in Adobe Photoshop to produce a single, fully focused image.

### Sample collection and preparation

All specimens were cleaned with acetone to remove possible contaminants (e.g. glue) before sampling. Enamel(oid) samples were extracted using a Proxxon GG12 hand-held drill equipped with a diamond-tipped bit (1.6 mm diameter). Powdered enamel(oid) samples were weighed for analysis.

### Oxygen and carbon isotope analysis in carbonates

Oxygen and carbon stable isotope analyses of the carbonate fraction were conducted on enamel(oid) (apatite ~ 500 µg) and the ammonite (CaCO_3_ ~ 50 µg). Samples with sufficient enamel(oid) (~ 2000 µg) were pretreated using 1.7% NaOCl to remove organic material and 0.1 M HAc to dissolve secondary CaCO_3_ [15]. Leached and unleached specimens were compared to assess diagenetic alteration. Analyses took place at the Vrije Universiteit Amsterdam (VU) using a GasBench II coupled to a ThermoFinnigan DeltaPlus IRMS [16]. CO_2_ was liberated by reacting the samples with water-free phosphoric acid (100% H_3_PO_4_) under a constant temperature of 45 °C. This reaction took place in exetainer vials that had been flushed with helium to remove atmosphere. The reaction times were 1,5 h for CaCO_3_, and 24 h for apatite. The resulting CO_2_ was analysed from the headspace, taking 10 subsamples from every vial. The data of the last eight measurements was used to calculate robust isotopic means. A CO_2_ monitoring gas with a constant isotopic ratio was used during the measurement to determine the δ^13^C and δ^18^O values of the samples relative to this gas. The isotopic composition (δ^13^C and δ^18^O) was calibrated using standards LSVEC, BCT (replacement for NBS19), and NBS18 [17]. If necessary, sample size corrections were applied using the in-house carbonate standard (VICS), which was analysed throughout each run. Attached sediment from the crocodylian jaw (NDGS 18199) yielded insufficient CO_2_ for reliable isotopic analyses. The δ^13^C and δ^18^O analysis of the *?Plioplatecarpus* tooth (NDGS 20303) failed due to a too high signal. The IAEA-603 that was used as a control standard for CaCO_3_, provides δ^13^C at + 2.46‰ and δ^18^O at -2.37‰, with standard deviations of less than 0.1‰ for δ^13^C and 0.2‰ for δ^18^O. For bioapatite samples, two in-house standards (horse enamel(oid) and horse dentine), as well as the inter-laboratorial Ag-lox (elephant tooth) standard, were employed, providing a reliable measure of reproducibility of apatite measurements, with long-term precision determined to be within 0.4‰ for both δ^13^C and δ^18^O [18]. The δ^13^C and δ^18^O are expressed against at the Vienna Peedee Belemnite (VPDB) scale. For comparison to the phosphate analyses, VPDB is converted into Vienna Standard Mean Ocean Water (VSMOW) [19].

### Oxygen isotope analysis in phosphates

Approximately 4 mg of leached enamel(oid) was used for converting the phosphate from fossil apatite to silver(ortho)phosphate (Ag_3_PO_4_). The enamel(oid) samples, along with NSB120c (phosphate rock) and Ag-lox standards, were weighed into 2 ml Eppendorf vials. The dry powder was dissolved in HF overnight and then neutralized with 25% NH_4_OH, with the aid of Bromothymol blue used as a pH indicator to adjust the solution to a pH of 7. An excess of 800 µl AgNO_3_ (2 M) was added to quantitatively precipitate Ag_3_PO_4_, instantly forming yellow crystals, which were rinsed multiple times with MilliQ water and dried overnight for analysis.

The oxygen isotope analysis of the silver phosphate was conducted at the VU using a DeltaPlusXP IRMS interfaced with a Finnigan TC/EA. Each sample was split into three subsamples of approximately 500 µg, tightly packed into silver capsules and converted into Ag_3_PO_4_ and stored in a desiccator under vacuum to avoid absorption of moisture. The glassy carbon reactor in the TC/EA was maintained at 1470 °C to convert the silver phosphate into CO, which was then directed through a gas chromatograph (GC) at 100 °C before isotopic analysis in the IRMS. Calibration of oxygen isotopic values was performed using the international USGS80 and USGS81 AgPO_4_ standards, included in each batch of 49 measurements to ensure accuracy and consistency with other laboratories. Additional controls (TU-1, TU-2 [20]), and VU internal standard phosphate VUSP (commercial Ag_3_PO_4_) were used as a quality control and (if necessary) to correct for drift.

### Strontium isotope analysis

Approximately 2 mg of tooth enamel(oid) and ammonite CaCO_3_ were subsampled for Sr isotope analysis. The pretreated material was dissolved in 0.5 mL of 3 M HNO₃ with the aid of an ultrasonic bath. Purified Sr fractions were extracted using Eichrom Sr-spec ion exchange resin and 1 drop of 0.5% H₃PO₄ was added and dried down. Hereafter, concentrated HNO₃ was added twice and dried down. The ^87^Sr/^86^Sr ratios were determined using a Thermo TRITON Plus TIMS at VU, with 200 ng of Sr loaded onto Re filaments, and TaCl₂ used as an activator. Ratios were corrected for mass fractionation, assuming an ^86^Sr/^88^Sr ratio of 0.1194, and normalized to the NBS987 standard (0.710245). Regular measurements of this standard yielded an average ^87^Sr/^86^Sr ratio of 0.710260 ± 1.5 · 10⁻⁶ (1σ, *n* = 31). Procedural blanks typically contain 35 pg or less of Sr, which is negligible for the analysis.

## Results

SYSTEMATIC PALEONTOLOGY

REPTILIA Linnaeus 1758 [21]

SQUAMATA Oppel 1811 [22]

MOSASAURIDAE Gervais 1853 [23]

MOSASAURINAE Gervais 1853 [23]

PROGNATHODONTINI indet. Russell 1967 [24]

*Referred Material*: NDGS 12,217: Isolated tooth crown, most likely derived from the posterior portion of the jaw.

*Description*: The crown of NDGS 12217 measures 29.3 mm in height, with a base 17.5 mm long anteroposteriorly and 11.4 mm wide labiolingually, giving it a laterally compressed, ‘D-shaped’ appearance in occlusal aspect and an ellipsoid base shape in basal aspect (Figure 2e). The upper 4 mm trending towards the apex is worn smooth, and the surface is covered with fine ridges and wrinkles which contribute to a distinctive veined texture. The strong bicarinate keels are positioned along the anterior and posterior margins, though the anterior carina is slightly offset and does not reach the tip, likely due to wear or chipping (Figure 2-a, b,e,) [25]. The carinae lack serrations, but the worn apices suggest small serrations may have been present. The apex is blunted, dome-shaped, and shifted posteriorly, giving the crown a slightly recurved appearance in lateral view with slight swelling near the base of the posterior margin (Figure 2-a). Typically, the more broadly rounded surface indicates this tooth may be from either a lower left or upper right position [26]. The aspect ratio of 1.6 in lateral view suggests it was shed from a posteriorly positioned tooth locus, like those found in *Prognathodon solvayi* [IRSNB R33,] [27], *P. sectorius* [NHMM 1998141] [28] and *P. kiandia* [MGUAN PA 129] [29]. The root lacks the periodontal ligament (PDL), confirming that the tooth was shed and not broken from the jaw pre- or postmortem. Although mosasaur teeth are typically known to have PDL [30], absence of PDL occurs more often [31, 32] and replacement teeth in some instances also form within the base of the functional tooth, and therefore upon shedding these teeth have no PDL [31]. A smooth scalloping along the conjuncture of the root and crown forms the ellipsoid concave interior indicating shedding [31, 33, 34]. The well-preserved texture and condition suggest minimal postmortem transport or reworking from older rocks like those in the Fox Hills Formation.

*Taxonomic Affinities of NDGS 12217 to Non-Mosasaurs*: Because NDGS 12217 was derived from a multi-taxic bonebed located in a geographic region where megatheropod and eusuchian fossils have also been collected, we evaluated the autapomorphic characteristics of the co-occurring taxa to exclude potential misidentifications (Table S1). First, NDGS 12217 was found near a large tyrannosaurid (cf. *Tyrannosaurus*) tooth. NDGS 12217 lacks denticles forming prominent serrations on the carinae, and has neither fluting nor longitudinal undulations, nor the traditional pachydont profile with split mesial carinae [35, 36], ruling out its identification as a tyrannosaurid tooth (also confirmed by T. Carr, pers. comm, 2023). An expanded referral to Paravians (Dromaeosauridae and Troodontidae) were also rejected as NDGS 12217 lacks their characteristic blade-like or lanceolate morphology with fluting and denticles [37]. At least three commonly collected Eusuchian taxa from similar geographic and temporal contexts were also surveyed. These include two heterodonts (*Brachychampsa* [ROM 68491] and *Borealsuchus* ([ROM 1903]), and one gavaloid (Thoracosaur; RSKM P3244.1 [38, 39]), all of which deviate notably from NDGS12217. Superficially, NDGS 12217 is similar to a fourth premaxillary tooth in adult *Brachychampsa* as seen in ROM 68491, and specifically more similar than the rather “Button-like” posterior teeth; however, the thicker enamel and only minor labial-lingual compression in ROM 68491 results in a more conical and bulbous crown that also lacks the more pinched carinae of NDGS 12217. Furthermore, it possesses a finely striated longitudinal texture rather than the distinct veined texture as seen in NDGS 12217. The overall size of the tooth of ROM 68491 is smaller when compared to NDGS 12217. In *Borealsuchus* (ROM 1903), the much weaker and finer striations coupled with the carinae differ from NDGS 12217. Moreover, *Borealsuchus* also possesses conical teeth along the concavo-convex margin of the jaw which differs from the ellipsoid cross section seen in NDGS 12217 and is not considered a good candidate for referral [39, also confirmed by D. D’Amore, pers. comm, 2025]. The slight mesiodistal smooth carina is not as pronounced in comparative thoracosaurs as in NDGS 12217, nor are they as compressed; in essence thoracosaur teeth are more gracile [40, also confirmed by A. Cossette, pers. comm, 2024]. The slightly ovate and tall caniniform shape and differing texture in RSKM P3244.1 also differs from NDGS 12217 [38]. Interestingly, as crocodilians mature, the teeth become more robust and conical from a lenticular shape; with a decrease in complexity of the enamel [41, 42]. NDGS 12217 would have to mature opposite in this series in order to form autapomorphies observed when compared to ROM 68491 despite different tooth positions. This would make NDGS 12217 an aberrantly large young eusuchian and implausible according to the body plan when compared to contemporaries of NDGS L4327 [42].

*Taxonomic Affinities of NDGS 12217 within Mosasaurs*: The dental morphology of NDGS 12217, particularly its veined texture, offset carinae, and basal cross section, confidently align it within the tribe Prognathodontini [1]. The tooth lacks the striations found in plioplatecarpines and tylosaurines, as well as the faceted morphology typical of *Mosasaurus* species, including *M. hoffmanni* and *M. conodon* [1, 25, 43–47]. Instead, it shares features with the species *Prognathodon solvayi*, *P*. *currii*, *P*. *giganteus*, and *P*. *staturator*, which also exhibits the diagnostic wrinkled and veined texture concentrated near the apex and inlaid with smoother enamel, blunted apexes, strongly bicarinate keels with varying levels of serrations [48, 49]. However, the exact species-level identification remains uncertain due to the insufficient overlap of deeper characters and limited material available, and this is why open nomenclature is being used (Table S1). Notably, NDGS 12217 also contains similarities to isolated teeth from European localities, such as the La Secca assemblage in Italy and the Stevns Klint Formation in Denmark that further support the referral to Prognathodontini [50, 51]. Additionally, the ‘Breien Mosasaur’ remains, discovered in 2016, also from the Hell Creek Formation, consisting of an articular-prearticular complex and a vertebra, further supports the presence of mosasaurine mosasaurs in the region during the latest Maastrichtian [5]. The ‘Breien Mosasaur’ most likely belongs to the same taxonomic cluster as NDGS 12217 due to their close stratigraphic and geographic association. While the fragmentary and isolated nature of both specimens prevents a definitive referral of the tooth to the same taxon, it does clarify and support a closer relationship of the two.

### Isotopic results

**Fig. 3:**
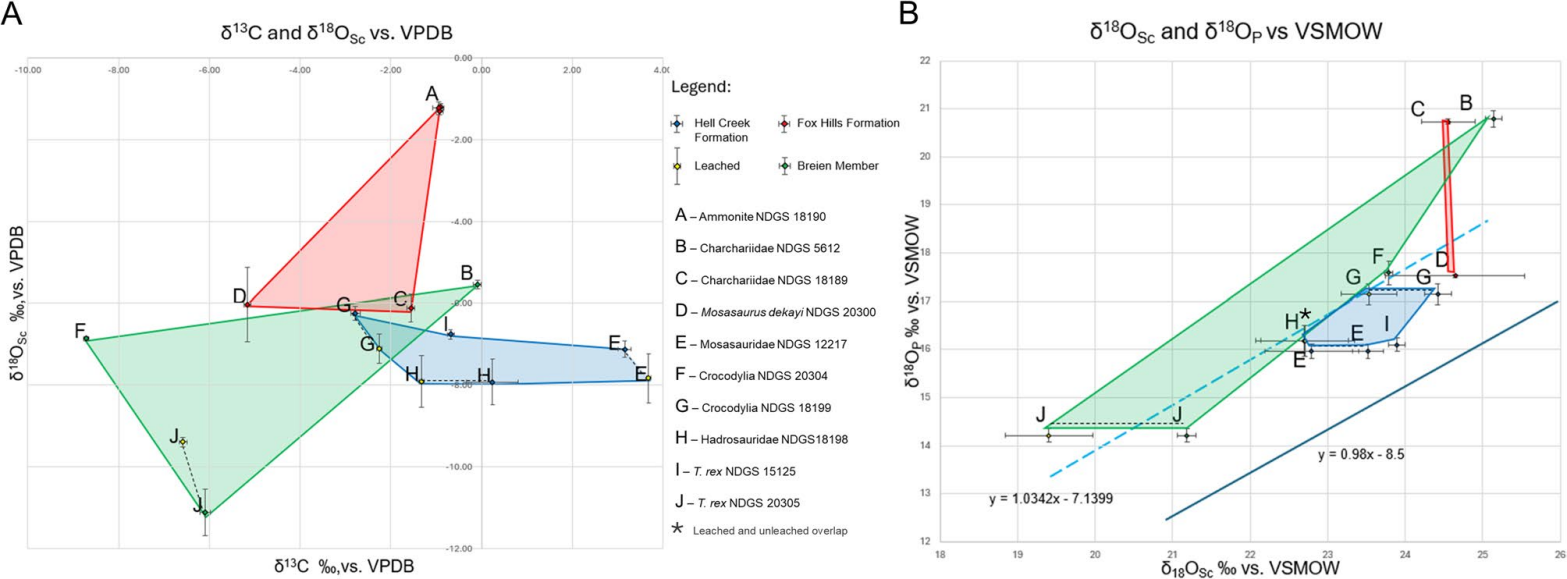
**a**: δ^13^C and δ^18^O_Sc_ with error margins for enamel(oid) apatite and ammonite calcite vs. VPDB. **b**: δ^18^O_Sc_ vs. δ^18^O_P_ for samples with both proxies. Dark blue line: theoretical δ^18^O_P_ - δ^18^O_Sc_ regression for mammals [46]; light blue dashed line: δ^18^O_P_ - δ^18^O_Sc_ regression for this study. Shaded areas: blue for the study site (NDGS L4327), green for Breien Member, red for Fox Hills Formation. Dashed lines connect leached (yellow) and unleached samples. Note that the δ^13^C and δ^18^O_Sc_ are obtained from apatite, except that of the ammonite

The stable isotope analyses of carbon and oxygen on leached and unleached enamel(oid) samples (Table 1; Fig. 3A) reveal mean deviations of -0.005‰ δ^13^C and − 0.82‰ δ^18^O_Sc_, probably indicative of some degree of diagenetic alteration [52], although it is also known that leaching could have adverse effects on modern biogenic apatite [53]. Taking these offsets into account, the δ^18^O_Sc_ and δ^13^C values span much broader ranges from − 11.11 to -1.20‰ and 3.68 to -8.72‰ vs. VPDB, respectively. Notably, the mosasaurine tooth NDGS 12217 yields 3.16‰ δ^13^C unleached, and 3.68‰ δ^13^C leached and − 7.12‰ δ^18^O_Sc_ unleached, and − 7.83‰ δ^18^O_Sc_ leached – which is consistently closer to terrestrial taxa than to marine, with the δ^13^C values exceeding beyond those observed in the terrestrial taxa and, therefore, potentially reflecting diet.

**Table 1:** All δ^13^C and δ^18^O_Sc_ vs. VPDB, δ^18^O_P_ ‰ vs. VSMOW, and ^87^Sr/^86^Sr data from this study. **Shaded areas** reflect leached samples. Error estimates are given by standard deviation (σ)

Reg. number	ID	Taxon	Locality	δ^13^C ‰ vs.VPDB		δ^18^O_Sc_ ‰ vs.VPDB		δ^18^O_*P*_ ‰ vs.VSMOW		^87^Sr/^86^Sr	
				mean	σ	mean	σ	mean	σ	mean	σ
NDGS 18190	A	Ammonoidae	NDGS L140	-0.92	0.07	-1.31	0.09	-	-	0.70774	6.69*10^6^
				-0.90	0.05	-1.20	0.08			0.70759	7.17*10^6^
				-0.95	0.12	-1.23	0.16			0.70761	6.55*10^6^
NDGS 5612	B	Charchariidae	NDGS L301	-0.09	0.09	-5.55	0.11	20.8	0.17	0.70772	6.70*10^6^
										**0.70775**	5.73*10 ^6^
NDGS 18189	C	Charchariidae	NDGS L140	-1.56	0.08	-6.12	0.34	20.73	0.05	0.70745	7.10*10^6^
										**0.70759**	6.70*10 ^6^
NDGS 20300	D	*Mosasaurus dekayi*	NDGS L148	-5.16	0.08	-6.03	0.90	17.52	0.03	0.70730	7.32*10^6^
										**0.70736**	7.01*10 ^6^
NDGS 12217	E	Mosasauridae	NDGS L4327	3.16	0.14	-7.12	0.20	15.97	0.16	0.70682	8.08*10^6^
										0.70683	7.28*10^6^
				**3.68**	**0.04**	**-7.83**	**0.60**			0.70684	6.97*10^6^
										**0.70689**	6.97*10 ^6^
NDGS 20304	F	Crocodylia	NDGS L2000	-8.72	0.06	-6.86	0.05	17.59	0.25	**0.70729**	7.85*10 ^6^
NDGS 18199	G	Crocodylia	NDGS L4327	-2.78	0.10	-6.25	0.17	17.15	0.21	0.70684	7.73*10^6^
										0.70686	7.49*10^6^
				**-2.25**	**0.06**	**-7.11**	**0.36**			**0.70689**	7.49*10 ^6^
NDGS 18198	H	Hadrosauridae	NDGS L4327	0.24	0.57	-7.92	0.56	16.19	0.32	0.70691	7.50*10^6^
										0.70691	7.27*10^6^
				**-1.32**	**0.05**	**-7.91**	**0.64**			**0.70704**	8.10*10 ^6^
NDGS 15125	I	*T. rex*	NDGS L4327	-0.68	0.08	-6.76	0.11	16.1	0.13	0.70689	1.58*10^5^
NDGS 20305	J	*T. rex*	NDGS L2000	-6.58	0.09	-9.39	0.12	14.21	0.13	**0.70691**	7.64*10 ^6^
				**-6.09**	**0.11**	**-11.11**	**0.57**				
NDGS 20301	K	Crocodylian	NDGS L148	-	-	-	-	19.9	0.18	-	-
NDGS 20303	L	*? Plioplatecarpus*	NDGS L142	-	-	-	-	16.81	0.07	0.70731	7.75*10^6^
										**0.70734**	6.83*10 ^6^

## Discussion

The published mosasaurine geochemical record from the Maastrichtian WIS does not contain δ^13^C and δ^18^O_Sc_ records. When comparing to the European records, NDGS 12217’s δ^13^C of 3.68‰ is significantly higher than typical marine mosasaurs, which range between −6.95 and −13‰ VPDB [28, 54]. Typically, δ¹³C values in tooth enamel(oid) rise by approximately 1‰ with each increase in trophic level [55–59]. However, other studies [54, 60] suggest that larger mosasaurs tend to exhibit lower δ¹³C values due to prolonged diving. Furthermore, they suggest that juvenile and subadult individuals likely occupied lower trophic levels and may have been restricted to nearshore environments. Tooth NDGS 12217, however, looks nothing like the juvenile *Plioplatecarpus* sp. from Stevns Klint [51]. NDGS 12217 is roughly 2.5 times larger, and its morphology agrees with adult teeth of *Prognathodon* [1, 48, 49], therefore, there is no reason to suspect it occupied lower trophic levels. The high δ¹³C values likely reflect a freshwater diet and potentially a higher trophic level [56] instead of lower. Consistent δ^18^O_Sc_ isotope signatures (-7.91 to -7.11‰) in NDGS L4327 teeth, alongside more variable δ^13^C values (-2.25 to 3.68‰), point to a shared water source or similar meteoric water uptake during the fossilization process [61].

Oxygen isotope measurements on the phosphate fraction (δ^18^O_P_), which are considered insensitive to abiotic diagenesis, also support this freshwater interpretation, with NDGS 12217 plotting closer to freshwater crocodylians and dinosaurs than to sharks. Comparison of δ^18^O_P_ and δ^18^O_Sc_ (Figure 3B) shows that none of the enamel samples plot directly on the theoretical of δ^18^O_P_- δ^18^O_Sc_ line, but shows a similar deviation to archosaurs, which may have a different linear relationship between δ^18^O_P_ and δ^18^O_Sc_ than mammals [62]. The δ^18^O_P_ values for NDGS L4327 teeth display a narrow range (15.97‰ to 17.15‰), contrasting the broader range in the Breien Member (14.21‰ to 20.80‰) and the Fox Hills Formation (16.81‰ to 20.73‰). All measured teeth in this study show similar or lower δ^18^O_P_ signatures compared to previously recorded freshwater excursions in other mosasaurs, such as *Pannoniasaurus* and *Goronyosaurus*, both of which adapted to non-marine environments [4, 10, 63].

**Fig. 4:**
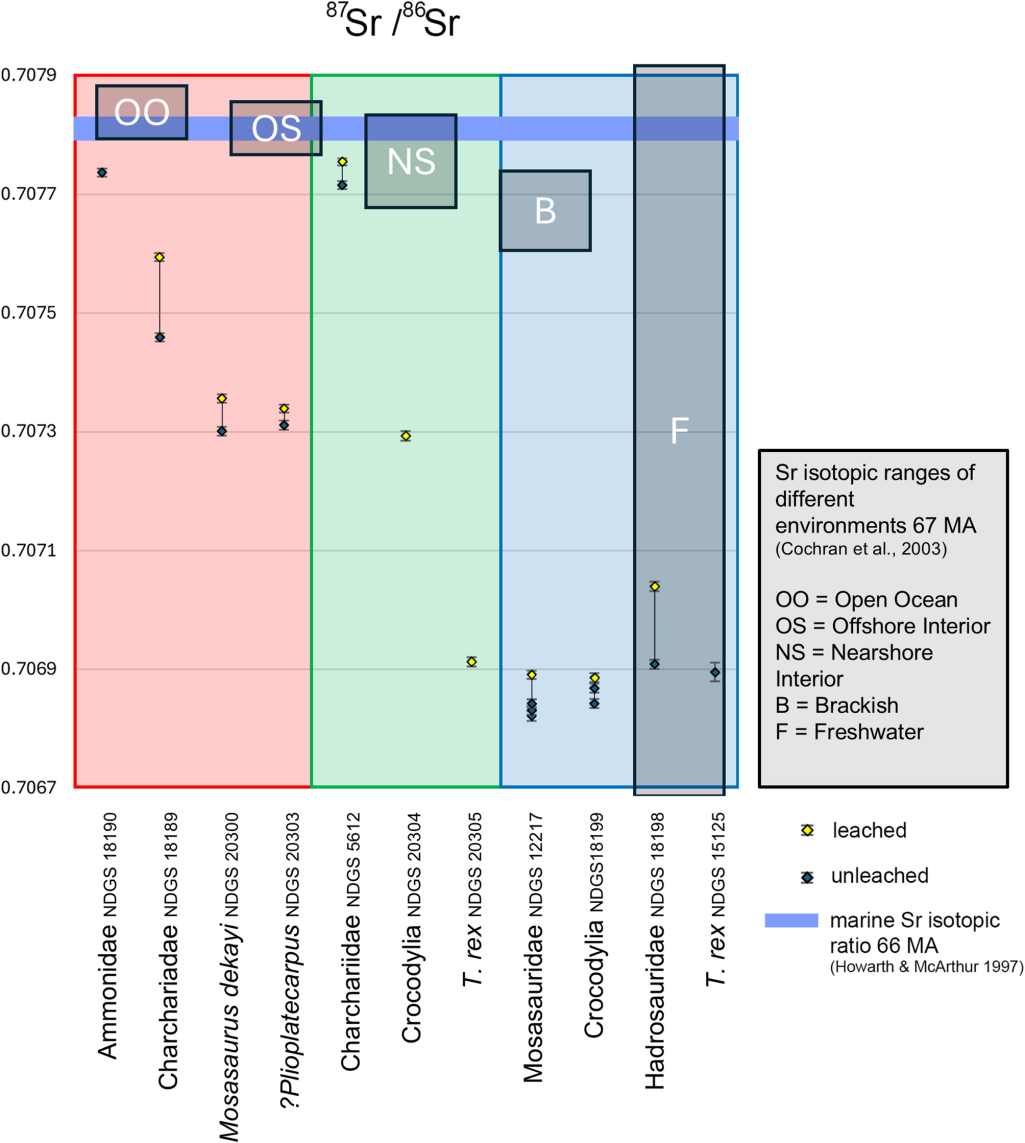
^87^Sr/^86^Sr ratios for all taxa compared to the marine isotopic ratio 66 million years ago [14]. Shaded areas: blue for the study site (NDGS L4327), green for Breien Member, red for Fox Hills Formation. Dashed lines connect leached and unleached samples; multiple analyses per specimen are plotted vertically

The ^87^Sr/^86^Sr ratios support a freshwater origin for NDGS 12217 (Fig. 4). Although the freshwater ^87^Sr/^86^Sr range is incredibly broad due to the variation in source-rock, the marine ^87^Sr/^86^Sr range is notably narrow (0.707839 ± 0.000024 average open ocean; through 0.707763 ± 0.00003014 average nearshore; [14, 64]). All measured teeth in this study yielded ratios (far) below the ranges for open ocean through to brackish ecosystems 66 million years ago [14, 65], indicating a fully freshwater environment for NDGS L4327, while the Fox Hills Formation and Breien Member show slightly elevated ratios but still fall below the brackish range [14] (Fig. 4). Notably all specimens that exhibit a ^87^Sr/^86^Sr freshwater range, required access to the surface for respiration, whereas the ammonite and shark specimens that exhibit more brackish ranges, do not. Therefore, we propose that mosasaurs resided in freshwater, while sharks and ammonites were present in the denser underlying sea water - suggesting the presence of a halocline -where freshwater resided on top of sea water [66].

The measured ^87^Sr/^86^Sr ratios indicate a gradual desalinization of the WIS. Recorded rising temperatures [13, 14], may have further facilitated mosasaurine species colonizing freshwater ecosystems [12], akin to how modern marine species spread to freshwater environments aided by rising temperatures [67, 68]. The consistent δ^18^O_Sc_, δ^18^O_P_, and ^87^Sr/^86^Sr ratios in the mosasaurian teeth analyzed in this study, suggest in vivo freshwater signals were preserved [69–71], despite potential minor diagenetic alterations, supporting the idea that mosasaurs, including NDGS 12217, exploited freshwater environments [4, 5, 11, 12].

## Conclusion

During the Late Maastrichtian, as WIS salinity levels dropped and rainwater dominated the basin, prognathodontin mosasaurs (NDGS 12217) and possibly *Mosasaurus*, likely adapted to freshwater environments like the fluvial paleoenvironment of the Hell Creek Formation. The isotopic evidence from NDGS 12217 suggests the tooth was formed while the mosasaur lived in a freshwater setting, reflecting a broader trend of mosasaurians adapting to decreasing salinity in the basin. This adaptation may indicate that large rivers of the Hell Creek Formation paleoenvironment could support large-bodied taxa, despite it being more likely for younger, smaller individuals to exploit these nearshore to riverine habitats [12, 31]. This adaptability may have been a key factor in their ability to thrive in various ecological niches during the Late Cretaceous. Future research, including more detailed isotopic analyses and comparisons with other mosasaur specimens, will be essential to further understanding the extent of this ecological flexibility.

## Supplementary Information

Below is the link to the electronic supplementary material.

Supplementary Material 1

## Data Availability

Data is provided within the manuscript or supplementary information files.
